# Snake venom VEGF Vammin induces a highly efficient angiogenic response in skeletal muscle via VEGFR-2/NRP specific signaling

**DOI:** 10.1038/s41598-017-05876-y

**Published:** 2017-07-17

**Authors:** Pyry I. Toivanen, Tiina Nieminen, Johanna P. Laakkonen, Tommi Heikura, Minna U. Kaikkonen, Seppo Ylä-Herttuala

**Affiliations:** 1 Department of Biotechnology and Molecular Medicine, A.I.Virtanen Institute for Molecular Sciences, University of Eastern Finland, Kuopio, Finland; 2 Heart Center and Gene Therapy Unit, Kuopio University Hospital, Kuopio, Finland

## Abstract

Vascular Endothelial Growth Factors (VEGFs) are promising molecules for the treatment of ischemic diseases by pro-angiogenic therapy. Snake venom VEGFs are a novel subgroup with unique receptor binding profiles and as such are potential new therapeutic agents. We determined the ligand-receptor interactions, gene regulation and angiogenic properties of *Vipera ammodytes* venom VEGF, Vammin, and compared it to the canonical angiogenic factor VEGF-A to evaluate the use of Vammin for therapeutic angiogenesis. Vammin efficiently induced VEGFR-2 mediated proliferation and expression of genes associated with proliferation, migration and angiogenesis. VEGF-A_165_ and especially VEGF-A_109_ induced less pronounced effects. Vammin regulates a number of signaling pathways by inducing the expression of NR4A family nuclear receptors and regulators of calcium signaling and MAP kinase pathways. Interestingly, MARC1, which encodes an enzyme discovered to catalyze reduction of nitrate to NO, was identified as a novel VEGFR-2 regulated gene. In rabbit skeletal muscle adenoviral delivery of Vammin induced prominent angiogenic responses. Both the vector dose and the co-receptor binding of the ligand were critical parameters controlling the type of angiogenic response from sprouting angiogenesis to vessel enlargement. Vammin induced VEGFR-2/NRP-1 mediated signaling more effectively than VEGF-A, consequently it is a promising candidate for development of pro-angiogenic therapies.

## Introduction

Vascular endothelial growth factors (VEGF) are key regulators of blood and lymphatic vessel formation and maintenance. They are potential gene candidates for therapeutic angiogenesis which aims to induce growth of new vasculature to ischemic tissues. To generate a sustained angiogenic response, delivery of the angiogenic factor by gene therapy vectors has been the favored approach. However, the completed phase II and III clinical trials have shown that a better understanding of VEGF biology and further development of the therapies are required for future clinical success^1^.

The VEGF family consists of five mammalian members VEGF-A, -B, -C, -D and placental growth factor (PlGF) as well as homologous proteins encoded by viruses (VEGF-E) and those found in snake venoms (VEGF-F). VEGFs mediate their signals through three VEGF receptor tyrosine kinases (VEGFR) -1, -2 and -3. The co-receptors, neuropilins (NRPs) -1 and -2 and heparan sulfate proteoglycans (HSPGs) modulate the functions of VEGFs. The VEGF family members are expressed as several isoforms, which differ in their HSPG and NRP binding properties^2^. The interactions of VEGFs with HSPGs mediate the formation of the VEGF concentration gradients required for proper vascular network formation during development^3^. VEGFR-1 binds VEGF-A with higher affinity than VEGFR-2, despite this VEGFR-2 is considered to be the main mediator of angiogenesis, as the kinase activity of VEGFR-1 is weak^4^. Nevertheless, VEGFR-1 has a crucial role during development as a non-signaling decoy receptor for VEGF-A^5^ and may also regulate the endothelial tip/stalk cell phenotype and sprouting by spatially sequestering VEGF from VEGFR-2^6, 7^. NRPs bind specific forms of several VEGF family members^8^ and are required for the normal development of the cardiovascular system^9^. NRP-1 has been shown to be involved in the control of endothelial tip/stalk cell phenotype^10^ and guidance of tip cells^11^. The mechanism of action of NRPs is still largely unknown, but recent studies have highlighted the importance of short motifs with C-terminal arginine for binding to NRP b1 domain^12^ and implied that NRPs can modulate VEGFR-2 downstream signaling by multiple mechanisms^2^.

Snake venom VEGFs form the most recently identified VEGF-F subgroup with varying receptor specificities^13^. Previous studies have demonstrated that they share the common structure with mammalian VEGFs^14^ and are able to induce vascular permeability^15^ and lower blood pressure^16^. However, their capability to induce angiogenesis *in vivo* has not been previously studied, even though their unique receptor binding profiles may have therapeutic advantages over mammalian VEGFs. Vammin is a novel VEGF-F family member isolated from the venom of sand viper *Vipera ammodytes* ^16^ and is a highly potent VEGFR-2 ligand devoid of VEGFR-1 binding and binds also to NRPs and HSPGs^17^.

The normal growth of the vascular tree requires temporally and spatially precise guidance signals mediated by the interactions of the VEGFs with their multiple receptors and co-receptors, which the current gene transfer techniques cannot easily mimic. A better understanding of the (co-)receptor-ligand interactions and the subsequent biological outcomes are required for the development of improved novel pro-angiogenic therapies. In this study, we: 1) characterized the ligand-receptor interactions and biological functions of Vammin; 2) evaluated the angiogenic potency of Vammin *in vivo* in rabbit skeletal muscle using adenoviral gene transfer and; 3) studied the contribution of VEGFR-1 and co-receptor binding to VEGFR-2-mediated angiogenesis *in vivo*.

## Results

### Receptor binding properties

**Figure 1:**
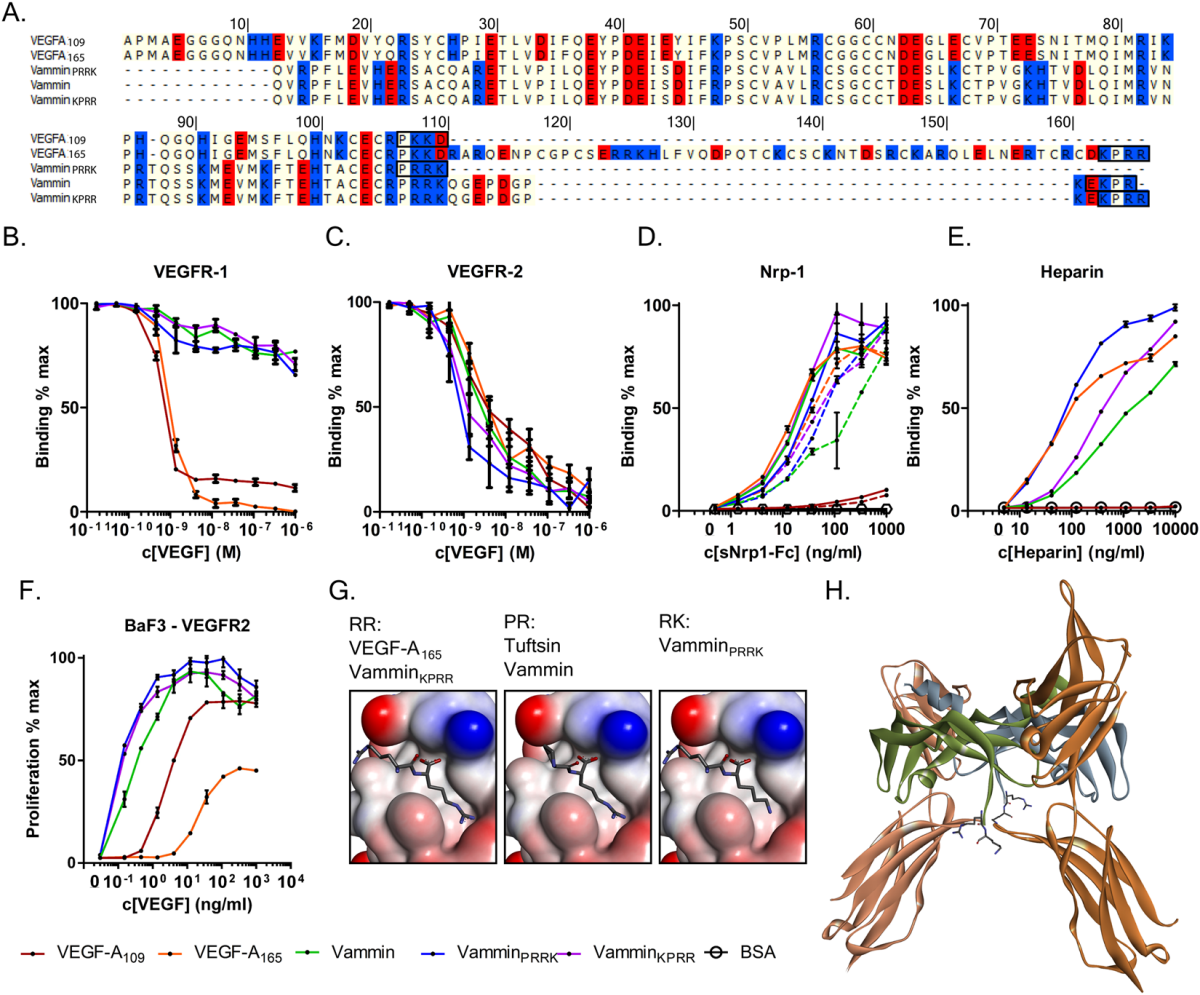
(**A**) Sequence alignment of recombinant VEGF-A and Vammin proteins. Basic amino acids are in blue and acidic in red background. The last four amino acids expected to be important for NRP-1 binding are in black boxes. (**B**) VEGFR-1 competition binding assay (**C**) VEGFR-2 competition binding assay (**D**) NRP-1 binding into VEGF coated plates the presence of heparin (solid lines) and without heparin (dotted lines) (**E**) Heparin binding in VEGF coated plates (**F**). BaF3-VEGFR-2 proliferation assay. In B to F the values are expressed as mean ± SD. (**G**) Binding of the two C-terminal amino acids into the NRP-1 binding pocket. RR and PR sequences are drawn based on VEGF-A_165_/NRP-1 and Tuftsin/NRP-1 complex structures. (PDB IDs 4DEQ and 2ORZ). RK sequence is modeled based on VEGF-A_165_/NRP-1 complex structure. Ligand amino acids are shown as sticks and NRP-1 as surface rendering colored by interpolated charge. (**H**) A model of location of the Vammin_PRRK_ C-terminus. Vammin crystal structure (PDB ID 1 WQ8) was superimposed onto VEGF-C/VEGFR-2 complex crystal structure (PDB ID 2X1W). The two VEGFR-2 monomers are seen with the brown colors and the two antiparallel dimer forming Vammin chains with green and blue. The C-terminal Arg97 and Lys98 of Vammin projecting outward from the complex are shown as sticks.

Competition binding assays were performed to characterize the binding of different VEGF-A forms and Vammin to VEGFR-1 and VEGFR-2. The VEGF-A forms were the only ligands binding to VEGFR-1 (Fig. 1B), whereas all tested factors bound to VEGFR-2 (Fig. 1C).

Ligand binding to NRP-1 b1 domain has been suggested to be dependent on C-terminal sequences resembling the VEGF-A exon 8 encoded C-terminus (CDKPRR) ending in free Arg. Previously, we showed that Vammin with a C-terminal sequence of PKEKPR binds to NRP-1^17^. In this study, we further characterized the NRP-1 binding site. Vammin_KPRR_, encoding 111 amino acids, was engineered to have an additional Arg residue in the C-terminus to mimic the last 4 amino acids (KPRR) in the C-terminus of VEGF-A. Vammin_PRRK_, encoding 98 amino acids, was designed to correspond to the VEGF homology domain encoded by VEGF-A_109_. Vammin_PRRK_ was expected not to bind to NRP-1 as it encodes a C-terminal Lys, instead of Arg, which was previously found to be a required binding determinant for NRP-1. VEGF-A_165_ which has the C-terminal NRP-1/HSPG domain and VEGF-A_109_ totally lacking this domain were used as controls (Fig. 1A). All ligands were equally efficient in VEGFR-2 binding (Fig. 1C). As expected, VEGF-A_109_ did not bind to NRP-1, in contrast all Vammin proteins and VEGF-A_165_ bound NRP-1 (Fig. 1D). As was previously shown^17^, free heparin increased the binding to NRP-1 (Fig. 1D), and accordingly, all Vammin forms and VEGF-A_165_ bound heparin (Fig. 1E). The C-terminal Arg of NRP-1 ligands, forms salt bridges with the NRP-1 binding pocket with both its side chain and the free carboxyl group, as shown in complex structures of both VEGF-A_165_, VEGF-C and a small peptide Tuftsin with NRP-1^12, 18, 19^. These bonds are modeled to be preserved also in Vammin_PRRK_ with the C-terminal Lys (Fig. 1G). In comparison, the C-terminal amino acid of VEGF-A_109_ is Asp with an opposite charge. Figure 1H shows a model of Vammin, containing amino acids up to the Lys 98, in complex with VEGFR-2. The Vammin C-terminus is located close to the VEGFR-2 domain 3 and projects outward from the VEGF/VEGFR complex. It is expected that VEGFR-2 and NRP-1 are able to bind simultaneously to form a signaling complex as shown previously^17^. The capability of the recombinant proteins to induce VEGFR-2 mediated cell proliferation was tested using VEGFR-2/EpoR expressing BaF3 cells (Fig. 1F). All three Vammin constructs were more potent and more effective in this assay than either of the VEGF-A forms.

### Vammin induces angiogenesis in rabbit skeletal muscles

**Figure 2:**
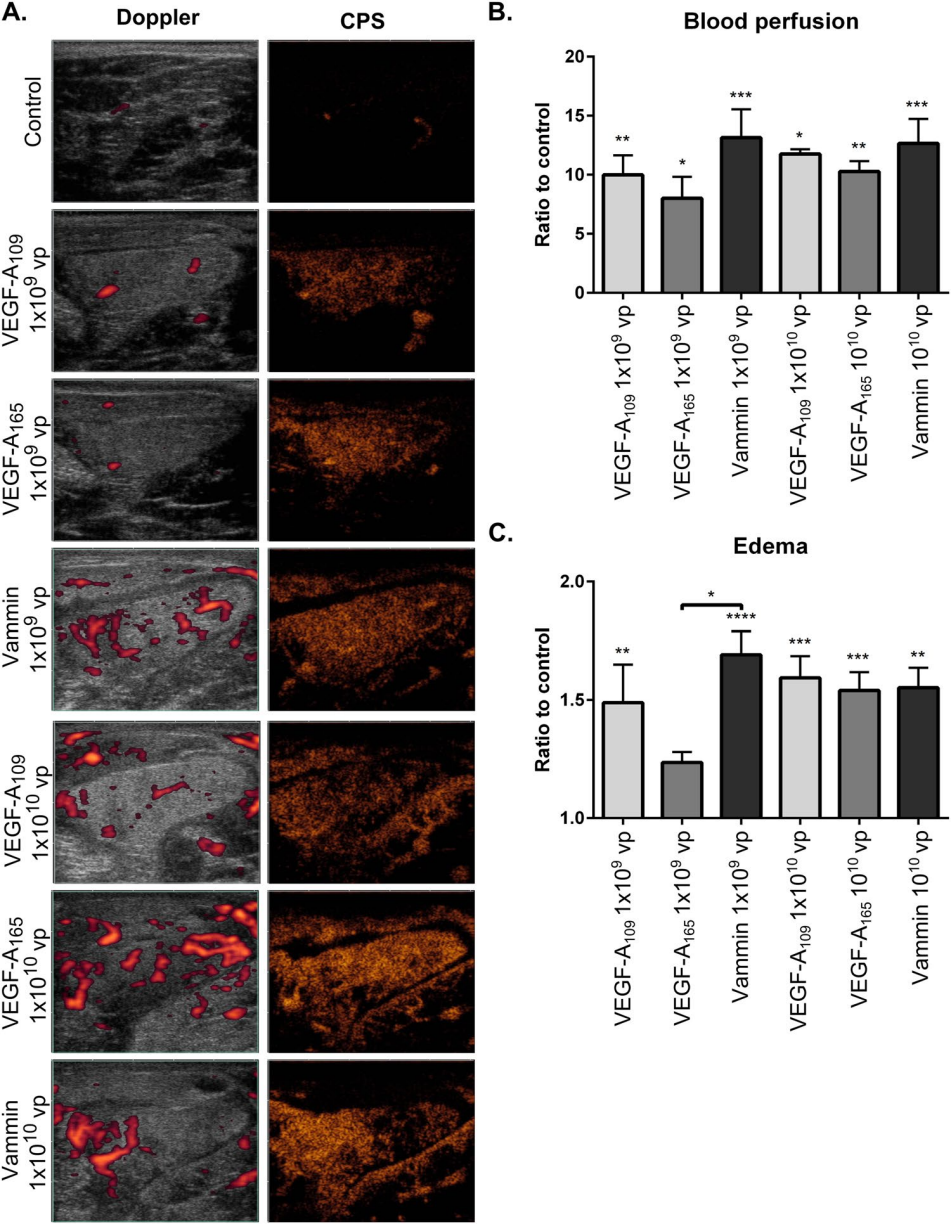
Vammin efficiently induces blood perfusion and edema in skeletal muscles. (**A**) Images with Doppler and CPS ultrasound from the semimembranosus muscles 6 days after the gene transfer. Increased perfusion is seen in all treated muscles. Edema is observed with both doses (**B**) Quantification of the blood perfusion from CPS ultrasound (n = 4–6 per group) (**C**) Quantification of edema from the ultrasound images (n = 4–6 per group). In B to C the data is presented as mean ± SEM. * P < 0.05, **P < 0.01, ***P < 0.001, ****P < 0.0001.

As Vammin was found to be a highly effective VEGFR-2 ligand, we generated an adenoviral vector encoding Vammin and compared its properties against vectors encoding either VEGF-A_165_, a VEGFR-1/VEGFR-2/NRP-1/HSPG ligand, or VEGF-A_109_ which is a VEGFR-1/VEGFR-2 ligand. Adenoviral vectors were injected into the rabbit hindlimb semimembranosus muscles in two doses, 1 × 10^9^ and 1 × 10^10^ viral particles (vp). A control vector (AdCMV) was used in three doses 1 × 10^9^, 1 × 10^10^ and 1 × 10^11^ vp. Blood perfusion in the target muscle was analyzed before and 6 days after the gene transfers using a contrast pulse sequence (CPS) ultrasound method, that is suitable for detecting blood flow in the capillary level vessels in addition to the larger vessels^20^. All three VEGF encoding vectors induced significant increases in blood perfusion (Fig. 2A and B). There were no significant differences in perfusion induced by the two different doses or between the different VEGF encoding vectors, but the higher doses were observed to cause increased perfusion also in the surrounding muscles, likely to be due to the spread of the growth factors and the vector. Edema was detected in the target muscles (Fig. 2A and C).

### Vector dose affects the vascular network morphology

**Figure 3:**
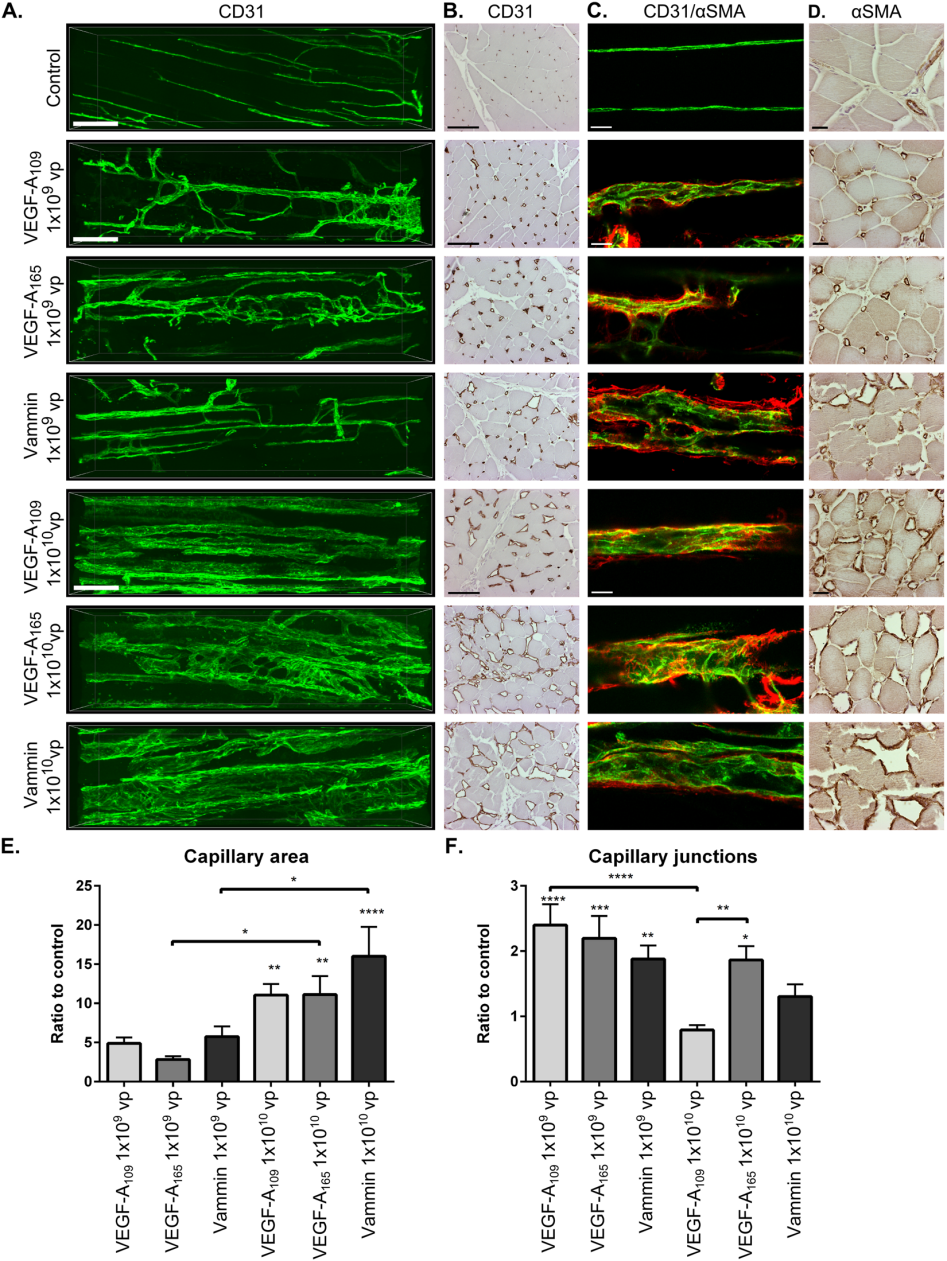
Both VEGF-A forms and Vammin induce dose and receptor binding profile specific changes in the vascular network (**A**). Multiphoton microscopy imaging of the blood vessel network by CD31 (green) labelled blood vessels from longitudinal sections of semimebranosus muscle. With 1 × 10^9^ vp dose all tested factors induce sprouting angiogenesis and modest enlargement of capillaries. With 1 × 10^10^ vp dose, the normal organization of the blood vessel network is disturbed. VEGF-A_109_ induces enlarged capillaries that have notably few connections and sprouts. Scalebar 100 µm. (**B**) CD31 staining of paraffin embedded cross-sections of semimembranosus muscle. Scalebar 100 µm. (**C**) Confocal microscopy images from CD31 (green) and αSMA (red) double-stained muscles. αSMA positive staining surrounds the enlarged capillaries. Scalebar 25 µm. (**D**) αSMA staining of the cross-sections shows high coverage of αSMA positive cells in the enlarged capillaries. In control muscles, the staining is mainly seen in the arterioles and venules. Scalebar 25 µm (**E**) Quantification of the capillary area as ratio to the controls from paraffin embedded CD31 stained cross sections (n = 4–6 per group) (**F**) Quantification of the amount of capillary junctions as a ratio to the controls from confocal microscopy images of CD31 stained whole-immunomounts (n ≥ 9 images per group). In E to F the data is presented as mean ± SEM. *P < 0.05, **P < 0.01, ***P < 0.001, ****P < 0.0001.

The blood vessel network in the affected area was imaged using confocal and multiphoton microscopy techniques from 1 mm thick longitudinal sections of the muscle. The two different vector doses showed highly different capillary tree morphology (Fig. 3A). With 1 × 10^9^ vp dose, the vessels were slightly enlarged and formed numerous connections in comparison to controls. With 1 × 10^10^ vp dose, all vessels were extensively enlarged (Fig. 3A,B and E) and fewer individual sprouts and connections were identified (Fig. 3A and F). VEGF-A_109_ induced uniform straight vessels running in a longitudinal direction through the muscle with very few connections. VEGF-A_165_ and Vammin caused a different vessel phenotype with enlarged vessels sending projections towards or fusing with adjacent vessels. At maximal affected areas, Vammin induced formation of CD31 positive vessels encircling the muscle fibers, whereas with VEGF-A_165_, individual sprouts were more clearly distinguished. Similar findings were seen in paraffin embedded cross-sections (Fig. 3B) where VEGF-A_109_-induced enlarged capillaries were homogenous in size and well separated from each other, whereas VEGF-A_165_ and Vammin induced highly enlarged vessels closely resembling the multiphoton imaging results.

### Cell recruitment after VEGF gene transfer and VEGF receptor expression

**Figure 4:**
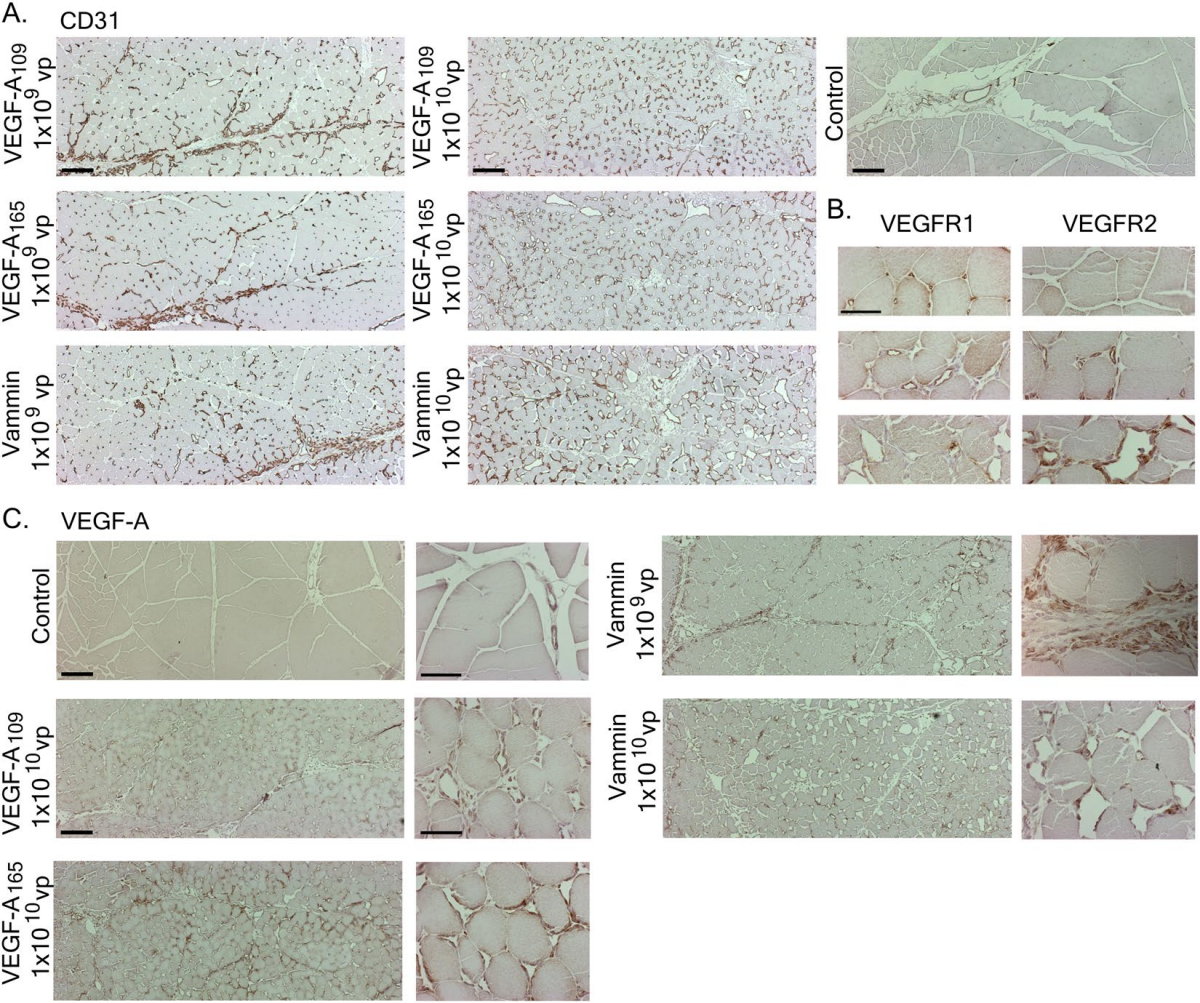
(**A**) Low magnification images from CD31 stained cross-sections of semimembranosus muscles show CD31 positive cells in the muscle perimysium in all 1 × 10^9^ vp animals. With the 1 × 10^10^ vp dose, enlargement of capillaries is uniformly seen inside the muscle fascicles. Scalebar 200 µm. B. VEGFR-1 staining is intense in the small capillaries, but reduced in the heavily enlarged capillaries. VEGFR-2 staining is more intense in the enlarged capillaries. Pictures are from AdVammin 1 × 10^10^ vp treated muscle from areas of low, medium and high effect. Scalebar 50 µm. C. Diffuse VEGF-A staining is seen in 1 × 10^10^ vp AdVEGF-A transduced muscles surrounding the capillaries especially in VEGF-A_165_ muscles. The cells in the perimysium were strongly stained with VEGF-A also in AdVammin 1 × 10^9^ vp transduced muscles suggesting that they may serve as a source of endogenous VEGF-A. VEGF-A is also detected around the highly enlarged capillaries in AdVammin 1 × 10^10^ vp muscle. Scalebar 200 µM in the low magnification (40x) images and 50 µm in the high magnification (400x) images.

The recruitment of mural cells was determined by staining cross-sections for αSMA. Very few positive cells were detected in control muscle capillaries with αSMA staining, and these were limited mainly to arterioles and venules, whereas almost all enlarged capillaries in growth factor treated animals were covered with αSMA positive cells (Fig. 3D). When imaged with confocal microscope from the longitudinal sections, αSMA staining was mainly localized in the immediate vicinity of the CD31 stained endothelium (Fig. 3C). As detected from low magnification images of the muscle cross-sections, the localization of the CD31 positive cells and capillary enlargement in the target muscle were different between the two vector doses. The 1 × 10^10^ vp dose caused a uniform effect covering the majority of the muscle area. In contrast, in the lower 1 × 10^9^ vp dose, the most dense CD31 positive staining was observed in the muscle perimysium, with CD31 positive cells packed around the edges of the muscle fascias (Fig. 4A).

VEGFR-1 staining was intense in the quiescent small capillaries of the controls and in areas with little capillary enlargement in VEGF samples (Fig. 4B). However, staining was notably weak in the strongly enlarged capillaries. On the contrary, VEGFR-2 staining was weak in the small capillaries, but intense in the enlarged capillaries. VEGF-A protein expression was detected in both VEGF-A_109_ and VEGF-A_165_ samples and surprisingly some also in Vammin samples (Fig. 4C), but not in the controls. The antibody used was verified to be able to detect VEGF-A_109_ and VEGF-A_165_ equally in both direct ELISA and western blotting, but showed no cross-reactivity towards Vammin in these assays. In the 1 × 10^9^ vp dose groups, the majority of the VEGF-A staining localized with the cells of the perimysium as shown with Vammin, whereas in samples from the 1 × 10^10^ vp dose, intense VEGF-A staining was found between the muscle fibers in the VEGF-A_165_ samples and less intense in the VEGF-A_109_ samples. Some VEGF-A staining was also detected in the strongly enlarged capillaries of the Vammin samples.

### Gene regulation in endothelial cells

**Figure 5:**
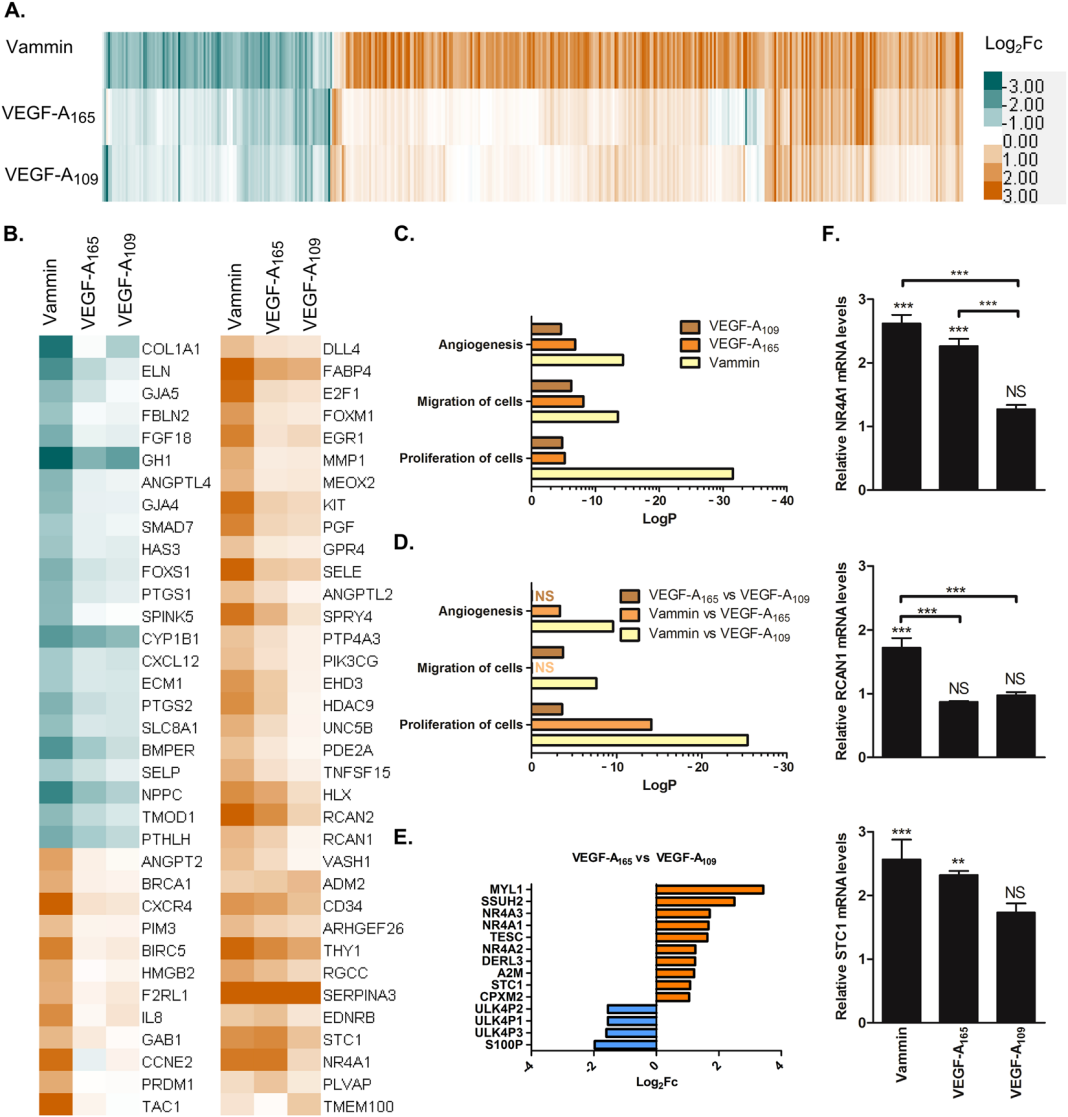
(**A**) Heatmap of fold changes (Log_2_) in gene expression levels between vectors encoding the tested factors and the AdCMV control vector. (**B**) Heatmap of fold changed (Log_2_) of the angiogenesis associated genes identified in the gene ontology analysis. (**C** and **D**). Gene ontology analysis using the Ingenuity Pathway Analysis: The top 3 most significantly enriched physiological functions are presented. (**E**) The genes differentially expressed between VEGF-A_165_ and VEGF-A_109_. F. qRT-PCR analysis of the expression of NR4A1, RCAN1 and STC1 in human dermal blood endothelial cells. The data is presented as mean ± SEM. **P < 0.01, ***P < 0.001.

To investigate the molecular mechanisms behind the observed differences in biological functions of the different tested factors, we stimulated HUVECs with adenoviral vectors encoding the tested factors and quantitated differential gene expression by RNA-sequencing. In general, the gene expression profiles were similar between all tested factors (Fig. 5A). We found 410 significantly differentially regulated genes with Vammin, 65 with VEGF-A_165_ and 28 with VEGF-A_109_ when compared to the control AdCMV vector (Fig. 5B and Supplementary data). In gene ontology analysis, Vammin and both forms of VEGF-A differentially regulated genes that were significantly associated with proliferation of cells, migration of cells and angiogenesis (Fig. 5B and C). When compared to VEGF-A_165_, Vammin induced significant differences in the expression of genes associated with proliferation and angiogenesis, but not migration (Fig. 5D). 14 genes were significantly differentially expressed between VEGF-A_165_ and VEGF-A_109_ (Fig. 5E), most notably NR4A1, NR4A2 and NR4A3 transcription factors, previously associated with VEGF-A_165_ signaling^21^.

Vammin regulated the expression of several genes encoding major angiogenesis contributing factors. ANGPT2 (Angiopoietin II), ANGPTL2 (Angiopoietin like II), DLL4 (Delta like ligand 4), PGF (PlGF) and VASH (Vasohibin 1) were upregulated whereas ANGPTL4 (Angiopoietin like IV), BMPER (BMP binding endothelial regulator) and CXCL12 (Stromal cell derived factor 1) were downregulated. Vammin also upregulated cell surface receptor encoding genes KIT, UNC5B and F2RL1 that has recently been shown to function as a switch between angiogenesis and vessel maturation depending on the cellular localization^22^. Of potential controllers of cellular signaling, GAB1 and four Sprouty family members controlling MAPK pathway signaling and Ca^2+^- Calcineurin-NFAT pathway signaling regulators RCAN1 and -2 and TESC were upregulated. Other potential new angiogenesis controlling signaling mediators included PALD1 phosphatase which was recently shown to be expressed in developing vasculature^23^ and kinase PBK which was found to phosphorylate p38MAPK in proliferating cells^24^. Interestingly, MARC1 gene encoding an enzyme catalyzing nitric oxide (NO) formation by nitrite reduction^25^ was upregulated by both VEGF-A_165_ and Vammin. To verify the high efficacy of Vammin to induce angiogenic signaling we stimulated human dermal blood endothelial cells (HDBEC) with the adenoviral vectors and using qPCR analyzed the expression of known VEGF regulated genes STC1^26^, RCAN1^27^ and NR4A1^21^. These genes were all significantly upregulated by Vammin (Fig. 5F).

## Discussion

The purpose of this study was to characterize the properties and angiogenic potential of a novel snake venom derived factor Vammin. To put the effects into context, we compared Vammin to two forms of a well characterized pro-angiogenic molecule VEGF-A. Additionally, we were interested in how the differential binding of the tested factors to VEGFR-1, NRP-1 and HSPGs affects the angiogenic responses.

NRPs have emerged as important contributors to angiogenesis, but their mechanism of action has remained elusive. A common feature of most NRP binding proteins is a C-terminal free Arg residue. However, the C-terminally modified Vammins showed that NRP-1 binding does not strictly depend on the C-terminal Arg residue since a related basic residue Lys also facilitated binding and is compatible with the NRP binding pocket. This is supported by earlier findings demonstrating that peptides with a C-terminal Lys could compete with VEGF-A_165_ for NRP-1 binding, albeit with lower efficacy compared to the ones with C-terminal Arg^28, 29^. In contrast, VEGF-A_109_ with C-terminal Asp is not capable of binding NRP-1 due to the opposite charge. The relatively relaxed binding determinants, binding motif which resembles the pro-protein convertase recognition site (R/K)X_n_(R/K) and the ability of several unrelated factors to bind^12, 30^ suggest that NRPs may have a role as modulators of many different signaling pathways. The use of both VEGF-A_109_ and VEGF-A_165_ in this study allowed us to evaluate potential genes specific for NRP in the context of VEGFR signaling. Notably, in comparison to VEGF-A_165_, VEGF-A_109_ failed to induce expression of NR4A1, NR4A2 and NR4A3 orphan nuclear receptors, which are regulated by the Ca-Calcineurin-NFAT signaling^21^. Additionally, other genes involved in calcium signaling including MYL1, TESC, STC1 and S100P were differentially regulated by these factors. This suggests that modulation of VEGFR-2 induced calcium signaling may be one important function of NRPs.

In the BaF3-VEGFR-2/EpoR proliferation assay, Vammin was a highly potent VEGFR-2 ligand when compared to either of the VEGF-A forms. Surprisingly, also VEGF-A_109_ was both a more potent and more efficient stimulator of these cells than VEGF-A_165_. Since the affinities of these ligands towards VEGFR-2 are comparable, this indicates that there are also other factors involved in determining the efficacy of signaling via VEGFR-2. A similar finding was previously shown for VEGFR-1 activation by VEGF-B and PlGF, which was affected by the differential orientation of the subunits of the dimerized receptor^31^. A similar phenomenon could also explain the observed differences of these factors in this assay. In HUVECs, Vammin induced a gene expression profile in line with a VEGFR-2 ligand activation. However, the changes were more prominent than with VEGF-A, especially Vammin induced the expression of genes linked to cellular proliferation. It is likely, that this is due to both the highly efficient VEGFR-2 activation and avoidance of VEGFR-1 binding, which limits VEGFR-2 mediated endothelial proliferation during embryonic angiogenesis^5^ and controls the amount of free VEGF-A available to signal through VEGFR-2^7^. However, it cannot completely be ruled out that some of the actions of Vammin could be mediated by yet unknown receptors on endothelial cells. Vammin induced expression of several genes known to contribute to angiogenesis including VEGF signaling targets NR4A1, RCAN1 and STC1. Additionally, several of the identified genes were involved in the regulation of MAP kinase pathway and Ca^2+^-Calcineurin pathway, both of which are important mediators of the VEGFR-2 signaling. Interestingly, MARC1, a gene encoding an enzyme recently discovered to catalyze NO reduction from nitrite^25^ was also upregulated. Given the importance of NO in VEGF signaling and the vascular system function, this might represent an alternative NO synthesis pathway to eNOS mediated NO generation from L-Arginine and may contribute to VEGF induced angiogenesis.

Adenoviral gene delivery to rabbit hindlimb verified that Vammin is a highly efficient angiogenic factor *in vivo*, greatly inducing blood perfusion in the target tissue. Whereas, the other VEGFR-2 specific ligand, VEGF-E, has been shown to induce less vascular permeability than VEGF-A^32^, Vammin induced at least as much edema as VEGF-A. The difference may reflect the lower VEGFR-2 binding affinity of VEGF-E^17^. Importantly, we found that even constant potent stimulation of VEGFR-2 over several days *in vivo* by Vammin does not lead to clearance of the receptor from the endothelium. The angiogenic effect of VEGFs can be divided into sprouting angiogenesis and capillary enlargement and the microenviromental concentrations of VEGFs are important for the type of angiogenic response^33^. Low doses of VEGF-A_165_ induce mainly sprouting angiogenesis and higher doses cause capillary enlargement and even formation of aberrant vascular structures^34^. This strict dose dependent effect of VEGFs applies also to both VEGF-A_109_ and Vammin. The tendency for sprouting angiogenesis with the low dose may be due to the gene transfer causing local hotspots of VEGF production, thereby generating intramuscular growth factor gradients. With the higher dose, the more efficient transduction causes more uniform production of VEGF around the muscle leading to saturated stimulus and mainly enlargement of the vessels. Accordingly, the high VEGF concentrations have recently been shown to synchronize endothelial cell function through Notch and VE-Cadherin mediated mechanism and to prevent sprouting^35^.

The major difference between the three ligands was the tendency of high dose VEGF-A_109_ to induce uniform formation of straight enlarged vessels with very few connections. This is in line with previous findings, showing that the interactions of VEGF-A with heparan sulphates and NRPs are required for sprouting angiogenesis during development^11, 36^ and may also reflect the attenuated response of VEGF-A_109_ seen in gene expression analysis. As both VEGF-A_165_ and Vammin are dual NRP-1/HSPG ligands, we can’t speculate which one of the interactions caused the difference in comparison to VEGF-A_109_. However, previous publications suggested that both may contribute^37^. In addition to the cell signaling mediated differences, the observed differential deposition and microenviromental concentrations of the heparin binding and non-binding factors are likely to be important. In addition to heparan sulphates, VEGFs also bind to several other extracellular matrix components^38^. These interactions and the difference in VEGFR-1 binding likely cause the subtle differences observed in vessel morphology with Vammin and VEGF-A_165_. The results suggest that modification of the ECM binding properties of VEGFs could be beneficial for developing VEGF based pro-angiogenic therapies. For example, fully soluble VEGF forms may be useful therapeutic agents to induce collateral growth by arteriogenesis with lesser disturbance of the capillary tree structure.

VEGFs are also known to be capable of recruiting bone marrow derived circulating cells that produce other angiogenic mediators and that may incorporate into the newly forming vessels^39^. A prominent feature after gene transfers was the presence of large amounts of CD31 positive cells in the muscle perimysium. These cells were determined to be a potential source for endogenous VEGF-A, which may have in part contributed to the angiogenic effect seen especially with the lower viral dose. Additionally recruitment of mural cells to the newly developed blood vessels is a critical step in the formation of stable vasculature. All three tested factors were efficient at recruiting αSMA positive cells into the enlarged vessels as was previously seen with VEGF-A_165_^40^. The lack of staining in the control muscle capillaries may reflect findings that αSMA is not expressed in quiescent pericytes and, therefore, its expression is induced only after angiogenic stimulus^41^. In the RNA-seq experiment, the tested factors did not directly upregulate genes linked to pericyte recruitment such as PDGF. Therefore, the stimulus may arise *in vivo* through increased blood flow and shear stress, rather than direct changes in VEGF induced gene expression.

This study sheds light into the interactions of NRP-1 with its VEGF ligands demonstrating the flexible binding determinants to NRP-1 and suggests that the NR4A nuclear receptors and calcium signaling may be important mediators of NRP-1 biological functions. Importantly, it demonstrates that the VEGFR-2 specific ligand Vammin is a highly potent molecule capable of inducing angiogenesis *in vivo* and warrants further studies in ischemic animal models of therapeutic angiogenesis. Binding to VEGFR-1 does not enhance the angiogenic response, but may rather limit the efficacy. This is in line with VEGF-D^ΔNΔC^ which despite its fairly low VEGFR-2 binding affinity is a potent angiogenic growth factor *in vivo* ^42^. The use of high doses of VEGF encoding gene transfer vectors for clinical applications comes with a risk of side effects due to vector escape into distant organs^34^. The high potency of Vammin to specifically stimulate VEGFR-2/NRP-1 signaling allows the use of very low doses of gene transfer vectors, and may therefore help to prevent systemic side effects. Alternatively, it may enable the use of different gene transfer vectors, with lower *in vivo* transduction efficacy, or cell type specific, but weaker, promoters, than the commonly used strong viral promoters.

## Methods

### Recombinant proteins and *in vitro* assays

The recombinant VEGF-A_109_, VEGF-A_165_, Vammin_KPRR_ and Vammin_PRRK_ proteins were produced in adherent HEK293T cells by calcium phosphate transfection with plasmids encoding N-terminally His-tagged proteins. On the following day, DMEM (Sigma-Aldrich) supplemented with 10% FBS was replaced with serum free media and cells were incubated for 2 days. The proteins were purified using HisTrapExcel columns with ÄktaAvant (GE Healthcare) using gradient elution from 0 to 500 mM Imidazole in PBS. The collected fractions were analyzed by SDS-PAGE. Pure fractions were pooled, diluted to PBS and concentrated using Amicon Ultra-15 3 K Centrifugal Filter Units and final buffer exchange to PBS was performed using HiTrap Desalting columns (GE Healthcare). Protein concentration was assessed using a BCA Protein Assay Kit (Thermo Scientific) and verified by SDS-PAGE. Recombinant Vammin, sVEGFR2-Fc and sNRP1-Fc were produced as described earlier^17^.

### Receptor binding assays

For VEGFR-1 and VEGFR-2 binding assays sVEGFR1-Fc (R&D Systems) and sVEGFR2-Fc proteins were pre-incubated for 1 h with dilution series of VEGF proteins in the presence of 100 ng/ml heparin (Sigma-Aldrich), the mixture was transferred to VEGF-A_165_ coated plates, blocked with 1% BSA in TBS, pH 7.5, for 2 h, and incubated for 1 h. For NRP-1 and Heparin binding assays 96-well plates were coated with the VEGF ligands overnight at pH 9.5 in bicarbonate buffer. Plates were blocked with 1% BSA in TBS, pH 7.5, for 2 h. sNRP1-Fc fusion protein and biotinylated heparin (Sigma-Aldrich) were incubated for 1 h at room temperature. The bound receptor-Fc fusion proteins and heparin-biotin were detected accordingly with anti-human IgG peroxidase (Sigma-Aldrich) or Streptavidin peroxidase and TMB (Sigma-Aldrich), respectively. Absorbances of stopped reactions were measured at 450 nm.

### Proliferation assay

BaF3-VEGFR-2 cells^43^ were cultured in DMEM supplemented with 10% FBS, 1% Penicillin-Streptomycin, 500 µg/ml G418 (InvivoGen) and 2 ng/ml rmIL3 (Calbiochem). Proliferation assay was performed by plating the cells in culture medium without rmIL3 at 18 000 cells per well and adding the indicated amounts of the ligands. Cells were incubated for 2 days and proliferation was measured using Cell Titer 96 Aqueuous One Solution Assay System (Promega).

### Adenovirus generation

Replication-deficient E1-E3 deleted clinical GMP-grade adenoviruses (serotype 5) were produced in 293 cells. Empty vector containing the CMV promoter and poly(A) tail was used as a control virus. All viruses used in the present study were tested for sterility, mycoplasma and endotoxin, and functionality in cell cultures before the animal studies.

### Gene transfers

All animal experiments and procedures used in this study were approved by the National Animal Experiment Board in Finland and carried out in accordance with the EU Act on the Protection of Animals Used for Scientific or Educational Purposes (497/2013) and The Finnish Act on Animal Experimentation. Animals were kept in standard housing conditions in The National Laboratory Animal Center of The University of Eastern Finland. Diet and water were provided ad libitum. New Zealand White rabbits received intramuscular injections of adenoviral vectors encoding Vammin (1 × 10^9^ and 1 × 10 × 10^10^ viral particles [vp]), VEGF-A_109_ (1 × 10^9^ and 1 × 10^10^ vp) or VEGF-A_165_ (1 × 10^9^ and 1 × 10^10^ vp) or CMV promoter (1 × 10^9^ and 1 × 10^10^ and 1 × 10^11^ vp) into the semimembranosus muscle of the thigh (n = 34). Rabbits were anesthetized with medetomidine (Domitor, 0.3 mg/kg, Orion) and ketamine (Ketalar, 20 mg/kg, Pfizer) and intramuscular gene transfers were performed in ten x 100 µl aliquots. One rabbit receiving VEGF-A_109_ 1 × 10^10^ vp gene transfer died at day 6 before ultrasound analysis and was excluded from analysis.

### Ultrasound analysis

Contrast pulse sequence (CPS) ultrasound imaging was performed before and at d6 after gene transfer for the analysis of changes in perfusion in the treated muscles as described previously ^20^. Acuson Sequoia 512 and 15 L8 transducer (Siemens AG, Erlangen, Germany) with Sonovue (Bracco, Milano, Italy) contrast agent were used to measure the perfusion in the muscles and the peak intensities were analyzed. One rabbit receiving VEGF-A_109_ 1 × 10^10^ vp gene transfer did not generate reliable contrast pulse sequence (CPS) response due to technical error and was excluded from CPS analysis. Other analyses were normally performed from this animal. Edema was determined as ratios of the area of the semimembranosus muscle before and at d6 after the gene transfer in the ultrasound figures.

### Immunohistochemistry

Tissues were fixed in 4% PFA, embedded in paraffin, and sectioned at 4–7 µm thickness. Immunostainings were performed using the following antibodies: α-SMA (1:500, Sigma-Aldrich, Cat. no A5228), CD31 (1:50, Dako, Cat. no M0823), VEGFR-1 (1:250, Santa Cruz Biotechnology, Cat no sc-31173), VEGFR-2 (1:250, Santa Cruz Biotechnology, Cat. no sc-6251) and VEGF-A (1:500, Santa Cruz Biotechnology, Cat no sc-7269). Photographs were taken with Olympus AX70 microscope (Olympus Optical, Tokyo, Japan). For whole-mount tissue imaging, 1 mm thick longitudinal sections were cut and stained with CD31 (1:100, Dako, Cat. no M0823) and α-SMA conjugated with Cy3 (1:500, Sigma-Aldrich, Cat. no C6198). For CD31, goat anti-mouse Alexa 488 (1:500; Invitrogen, Cat. no A-11001) was used. Imaging was performed by Nikon A1R multiphoton microscope (MPLSM) and LSM700 Zeiss confocal microscope (LSM).

### Analysis of gene expression

Human umbilical vein endothelial cells (HUVECs) were isolated from umbilical cords obtained from the maternity ward of Kuopio University Hospital. Informed consent was obtained from all the umbilical cord donors. All experiments were carried out in accordance with the European Union guidelines and regulations with the approval by the Kuopio University Hospital Ethics Committee (license numbers 58/2012 and 341/2015). Human dermal blood endothelial cells (HDBECs) were obtained from PromoCell (Heidelberg, Germany). Cells were used at early (I-V) passages and grown on plastic surface coated with 0.05% gelatin/10 µg/ml fibronectin (Sigma) in EBM Endothelial Cell Basal Medium supplied with EGM SingleQuots (Lonza). After 24 h of adenoviral transductions, starvation medium (EBM + 0.5% FBS) was changed to the cells. After another 24 h, RNA was extracted with RNeasy Mini Kit (Qiagen) accrording to the manufacturer’s instructions.

For RNA sequencing RNA was enriched for Poly(A)-RNA with MicroPoly(A) Purist Kit (Life Technologies). RNA was fragmented using RNA Fragmentation Reagents (Life Technologies) and purified by running through P-30 column (Bio-Rad, Hercules, CA, USA). Fragmented RNA was dephosphorylated with PNK (New England Biolabs, Ipswich, MA, USA) followed by heat-inactivation. Dephosphorylation reactions were purified using RNA Clean & Concentrator™-5 kit (Zymo Research). Poly(A)-tailing and cDNA synthesis was performed as described^44^. However, for reverse transcription an oligo allowing custom barcoding during final amplification was used: /5Phos/GATCGTCGGACTGTAGAACTCTGAAC/iSp18/TCAGACGTGTGCTCTTCCGATCTTTTTTTTTTTTTTTTTTTTVN (IDTDNA). After cDNA synthesis, Exonuclease I (New England Biolabs) was used to catalyze the removal of excess oligos. The DNA-RNA hybrid was purified using ChIP DNA clean & Concentrator Kit (Zymo Research Corporation, Irvine, CA, USA), RNaseH treated and circularized using CircLigase (EpiBio). The libraries were amplified for 11 cycles using the following primers:

5′-AATGATACGGCGACCACCGACAGGTTCAGAGTTCTACAGTCCGACG-3′ and 5′-CAAGCAGAAGACGGCATACGAGATXXXXXGTGACTGGAGTTCAGACGTGTGCTCTTCCGATCT (barcode XXXXXX-underlined). The final product was ran on Novex 10%-TBE gel, purified and cleaned as above. The libraries were sequenced on HiSeq 2000 for 50 cycles according to the manufacturer’s instructions.

Two independent experiments were performed with the 1^st^ consisting of AdCMV and AdVammin, AdVEGF-A_109_ and AdVEGF-A_165_ as two replicates and the 2^nd^ consisting of AdCMV, AdVammin, AdVEGF-A_109_ and AdVEGF-A_165_ and AdVammin + AdCMV as two replicates. Data analysis was performed using HOMER 4.3 and the detailed instructions for analysis can be found at http://homer.salk.edu/homer ^45^. RNA-Seq was mapped using tophat allowing up to two mismatches and reporting only one alignment for each read. Poor quality reads were filtered out (minimum 97% of bp over quality cutoff 10). Each sequencing experiment was normalized to a total of 10^7^ uniquely mapped tags and visualized by preparing custom tracks for the UCSC Genome browser. Differentially expressed genes were identified using edgeR^46^, thresholds of FDR <0.05, reads per kb per million reads >0.5 and fold change >2 were used. Hierarchical clustering of the log_2_ fold-changes was performed by Cluster 3.0^47^ using Pearson correlation as distance method and average linkage as clustering method. The output from clustering was viewed using Java Treeview^8^. The gene ontology analysis was performed by Qiagen’s Ingenuity Pathway Analysis (IPA, Qiagen Redwood City, www.qiagen.com/ingenuity).

For the qRT-PCR the amount and purity of total RNA were measured with NanoDrop ND-1000 Spectrophotometer (NanoDrop Technologies Inc, Wilmington, DE, USA). One microgram of total RNA was reverse transcribed into cDNA using random hexamers (Thermo Scientific) and Revert Aid Reverse Transcriptase (Thermo Scientific). Quantitative measurements of mRNA levels were done using the Assays-on-Demand gene expression products (Life Technologies) with the StepOnePlus Real-Time PCR System (Life Technologies). Alternatively, Power SYBR Green PCR Master Mix (Life Technologies) and 10 pmol of primers were used (Supplemental Table I). Cycling parameters were 95 °C for 10 min, 40 cycles of 95 °C (15 s) and 60 °C (6fFor whole tissue lysate, respectively.

The RNA-seq data can be found under GEO accession number GSE68535.

### Statistical analysis

Results are expressed as the mean ± SEM. Statistical significance was evaluated with One-way ANOVA followed by Bonferroni’s corrected t-test. *P* < 0.05 was considered statistically significant.

## Electronic supplementary material

Supplementary Dataset 1

Supplementary Dataset 2

Supplementary Dataset 3

Supplementary Dataset 4

Supplementary Dataset 5

Supplementary Dataset 6
